# Demographic characteristics and neuropsychological assessments of subjective cognitive decline (SCD) (*plus*)

**DOI:** 10.1002/acn3.51068

**Published:** 2020-06-26

**Authors:** Lixiao Hao, Yu Sun, Yun Li, Jieyu Wang, Zichen Wang, Zhongying Zhang, Zhanyun Wei, Ge Gao, Jianguo Jia, Yue Xing, Ying Han

**Affiliations:** ^1^ Department of General Practice XuanWu Hospital of Capital Medical University Beijing China; ^2^ Department of Geriatrics XuanWu Hospital of Capital Medical University Beijing China; ^3^ Department of Neurology XuanWu Hospital of Capital Medical University Beijing China; ^4^ Department of General Surgery XuanWu Hospital of Capital Medical University Beijing China; ^5^ Radiological Sciences Division of Clinical Neuroscience Queen’s Medical Centre University of Nottingham Nottingham United Kingdom; ^6^ National Clinical Research Center for Geriatric Disorders Beijing China; ^7^ Center of Alzheimer’s Disease Beijing Institute for Brain Disorders Beijing China

## Abstract

**Background:**

Since SCD (*plus*) was standardized, little is known about its demographic characteristics and its outcomes of neuropsychological assessments, including the SCD questionnaire 9 (SCD‐Q9).

**Objective:**

To characterize SCD (*plus*) by comparing the neuropsychological features among its subgroups and with normal controls (NC). Also, to explore its demographics and to understand the relation of the chief complaints and the scores of SCD‐Q9.

**Methods:**

Multistage stratified cluster random sampling was conducted to select participants. As a result, 84 NC and 517 SCD (*plus*) were included. SCD (*plus*) was further classified into several subgroups (SCD‐C: concerned cognitive decline; SCD‐F: complaints about SCD within the past five years; SCD‐P: feeling performance being not as good as their peers; SCD+: presented> 3 of SCD (*plus*) features; SCD‐: presented ≤ 3 of SCD (*plus*) features (see the diagnostic criteria for the details)) and between‐group comparisons of neuropsychological scores were conducted. Point‐biserial correlation and binary logistic regression analyses were performed to investigate the demographic characteristics of its subgroups. Finally, Spearman correlation was used to better understand the relation of SCD (*plus*) to SCD‐Q9.

**Results:**

(1) Scores of AVLT‐LR (AVLT‐LR: Auditory Verbal Learning Test‐Long Delayed Recall) and MoCA‐B (MoCA: Montreal Cognitive Assessment‐Basic) were lower in the SCD‐P group than those in the NC group, and the SCD+ group scored lower in the MoCA‐B and CDT(CDT: Clock Drawing Test) than the SCD‐ group. (2) Females were more concerned than male participants. Individuals with lower education level felt that their cognitive performance were worse than their peers. Also, younger people might express concerns more than the more elderly. People who had complaints of SCD‐P might be more likely to report SCD‐C, but less likely to report SCD‐F. (3) Positive correlations were found between the chief complaints of SCD (*plus*) and some items of SCD‐Q9.

**Conclusions:**

SCD (*plus*) may be related to demographic factors. Individuals with SCD (*plus*) already exhibited cognitive impairment, which can be detected by SCD‐Q9.

## Introduction

As the most common neurodegenerative disorder, Alzheimer's disease (AD) has brought a huge economic burden to families and society.^1^ According to the National Institute on Aging‐Alzheimer Association (NIA‐AA) workgroups, the progression of AD can be divided into three different stages.^2^ Currently, no effective modifying therapy has been validated yet for stage II^3^ – mild cognitive impairment (MCI) and stage III^4^ – AD dementia. Thus, stage I, namely the preclinical AD stage may be the most important phase for potential effective intervention and therapeutic approaches.

Pre‐mild cognitive impairment subjective cognitive decline (pre‐MCI SCD), which has been defined as a self‐experienced persistent decline in cognitive capacity in comparison with a previously normal status and objective cognitive performance within normal ranges, is the first symptomatic manifestation of AD and has received increasing attention.^5^, ^6^, ^7^ Individuals present with the following specific manifestations associated with pre‐MCI SCD would be identified as SCD (*plus*) – a more severe condition of SCD: concerned about cognitive decline (SCD‐C); age at onset of SCD ≥ 60 years; complaints about SCD within the past five years (SCD‐F); the complainers feel their performance are not as good as their peers (SCD‐P); complaints were only limited in the memory rather than other cognitive domains (SCD‐M); a confirmed cognitive decline by the informants; and presence of the ApoE ε4 genotype and biomarker evidence for a potential progression to AD.^7^, ^8^ Early identification of the population with these disease characteristics is critical for possible early intervention of AD in the future.

According to the diagnosis framework, like normal controls (NCs), SCD (*plus*)’s objective neuropsychological assessments are within the normal range. However, the chance of SCD (*plus*) progressing to MCI or dementia was significantly higher than the NCs.^7^, ^9^ Therefore, it is of great significance to study the distinguishing features between the SCD (*plus*) and NC groups, which could be more practically important for identifying people with SCD (*plus*) at early stage and conduct early intervention. However, to the best of our knowledge, only very few studies have reported the objective cognitive assessment results of this particular population,^6^, ^8^ especially the SCD (*plus*) subgroups, such as SCD‐F and SCD‐P.

Additionally, even among the few studies that did report the relation between objective cognitive decline and subjective cognitive complaints in SCD (*plus*),^10^, ^11^ inconsistent findings were presented. These discrepancies may be due to different demographics in different cohorts.^12^ Previous evidence showed that the prognosis of SCD (*plus*) differed in different demographic subpopulations.^13^, ^14^ For instance, a few studies have reported a gender effect on the prevalence of SCD (*plus*)^6^, ^13^ – females are more inclined to report SCD‐C, both of which implied that gender might be an indicator of rapid conversion from SCD (*plus*) to MCI or AD, and yet little is known about the situation in China. Moreover, most of the studies on demographic characteristics focused on the SCD, and little is known about *them* for SCD (*plus*), which is a more advanced phase of subjective cognitive decline. Wang et al.^15^ and Snitz et al.,^16^ proposed that concerns about memory typically increase as individuals grow older and lower level education was related to more concerns about SCD.^17^, ^18^ Furthermore, other than SCD‐C, other chief complaints of SCD (*plus*), including SCD‐F and SCD‐P have not been studied.

Finally, SCD complaint is a global issue and due to its increased risk of progressing to MCI,^6^ various questionnaires have been developed to better identify early AD patients,^19^, ^20^, ^21^ such as the SCD‐questionnaire 9 (SCD‐Q9),^19^ and the self‐reported top 10 screening items.^20^ However, to our best knowledge, no study has reported the correlation and conformity between the chief complaints of SCD (*plus*) and those questionnaires.

Therefore, our current study aims to (1) examine the neuropsychological assessment characteristics of SCD (*plus*) and evaluate whether its various symptom clusters (subgroups) differ in predicting the neuropsychological changes of SCD (*plus*), following the similar approach as in Sanchez‐Benavides et al^8^; (2) explore the demographics associated with SCD (*plus*) subgroups; and (3) understand the relation of SCD‐Q9 and the chief complaints of SCD (*plus*). The ultimate purpose is to characterize SCD (*plus*) to provide further information for its early identification and intervention.

## Materials and Methods

### Ethics statement

This study was approved by the medical ethics committee of XuanWu Hospital of Capital Medical University, Beijing, China. Written informed consent was obtained from either participants or their legally acceptable representatives.

### Participants

#### Subject recruitment

The details of the study including its purpose, procedure, and contact information were advertised at large‐scale gatherings and via broadcasting with the permission of and support from the councils of ShunYi District in Beijing, China. Voluntarily, people were asked for their consent to join the study.

#### Study procedure and subject selection criteria

A multistage stratified cluster random sampling design was used to select the subjects within the consented cohort. The investigation was performed from September to November 2016. All participants completed questionnaires via face‐to‐face interviews with our trained physicians (all with over 10 years of experience) mainly based on self‐reporting. This includes information on sociodemographic characteristics, social support, medical history, and lifestyles. Informants were also involved for reporting cognitive complaints, medical history, and up‐to‐date mental status. Two thousand six hundred and eighty‐nine individuals who fulfilled the primary inclusion criteria were enrolled in our study. Further screening based on the exclusion criteria was performed and finally the participants were asked to complete all the neuropsychological assessments and subjective cognitive decline interview (SCD‐I)^22^ listed below.

Inclusion criteria include: permanent residents (living in the target community for at least half a year), Han ethnicity, and ≥60 years old.

Exclusion criteria include: (1) untraceable residents; (2) registered but died; (3) with serious physical illness(es); (4) minority ethnics; (5) living in a nursing home; (6) had been diagnosed of dementia; (7) medical history of severe schizophrenia, moderate depression and anxiety, or other mental problems; (8) neurological diseases: cerebrovascular disease, encephalitis, brain tumors, brain trauma, epilepsy, Parkinson's disease, progressive supranuclear palsy, Huntington^’^s disease, hydrocephalus that could lead to cognitive decline; (9) metabolic diseases: anemia, thyroid dysfunction, lack of folic acid and vitamin B_12_; (10) a history of CO poisoning; (11) a history of general anesthesia; (12) severe problems of vision, hearing, or speaking, and because of these reasons, were not able to participate in the neuropsychological evaluation.

#### Assessment and diagnosis procedure

Subjects were required to complete the SCD‐Q9,^19^, ^23^ Hamilton Depression,^24^ and Anxiety Scale.^25^ The general neurological examination including assessments of the sensory neurons, motor responses, and reflexes was performed. Information about cognitive complaints, medical history, and up‐to‐date mental status was also collected. A comprehensive neuropsychological test battery contains the following four cognitive domains: (1) Memory: Auditory Verbal Learning Test Hua Shan‐(AVLT‐H)^26^; (2) Executive function: the Trail Making Test B (STT‐B)^7^; (3) Language: An Animal Fluency Test (AFT)^28^; and (4) Visual space function: the Clock Drawing Test (CDT‐30).^29^ These four domains plus Montreal Cognitive Assessment‐Basic (MoCA‐B)^30^ were used to evaluate the global cognition. The cognitive and functional performance related to the clinical stages of dementia was assessed using the Clinical Dementia Rating Scale (CDR).^31^ The Activity of Daily Living (ADL)^32^ was used to evaluate social functioning. The Hachinski Ischemic Scale (HIS)^33^ was performed to differentiate between degenerative and vascular etiologies. SCD‐I and scoring procedures as follows:

The SCD‐I allowed assessment of subjective cognitive decline in five different cognitive domains (memory, language, planning, attention, and any other cognitive decline).^18^ The interview consisted of three parts including an open question at the beginning as well as a structured part for the participant and the informant. In this study, we were mainly focusing on the structured part. For each domain, the physician asked the patient if he/she had noticed any worsening in function (e.g., “do you feel like your memory has become worse”). If the participant answered this question with yes, the physician added more in‐depth questions about the domain to assess the presence/absence of SCD (*plus*) features, that is, “Does this worry you?,” “How long ago did you start to notice the decline?,” and the performance in comparison to peers “Compared to other people of your age, would you say that your performance is worse?.” For detailed implementation process, see the appendix and the DELCODE study published in 2019.^22^

The ultimate cognitive diagnosis was determined by the expert panel. Based on the outcome of the above assessment, we finally classified participants into different groups as follows:

1. NC was assigned when participants did not have SCD complaints and achieved a normal score [>−1.5 standard deviations (SD) cutoff] in all four cognitive domains and MoCA‐B. Additionally, the CDR score of NC had to be zero.
2. For SCD (*plus*), all the following criterion needed to be met^7^: (1) participants reported the problem in memory; (2) age *of onset* ≥ 60 years old; (3) achieved a normal score in all four cognitive domains and MoCA‐B; (4) ADL was normal; (5) HIS score < 4.
3. Subgroups of SCD (*plus*): For SCD‐Cs, in addition to the SCD (*plus*) diagnosis criterion above, they also had *concerns* about their cognitive decline. SCD‐F and SCD‐P were similar to SCD‐C, but SCD‐F needed to have complaints about SCD within the past *five* years in addition. For individuals in the SCD‐P group, they all felt that their performance was not as good as their *peers* at a similar age.

Finally, to study the characteristics of SCD (plus) based on it severity, we grouped the population into SCD+ (more severe) and SCD− (less severe) subgroups. Given that all the subjects fulfilled a) reported memory problem and b) age of onset ≥60 years old, people in the SCD+ and SCD− subgroups had to present ＞1 or =1, respectively, of the three SCD (plus) feature as follows: (1) SCD‐C; (2) SCD‐F; (3) SCD‐P.

### Statistical analysis

We conducted all analyses using the Statistical Package for the Social Sciences version 17.0 (SPSS Inc., Chicago, IL). Descriptive statistics for scores of neuropsychological assessments, SCD‐Q9 and the proportion of SCD (*plus*) were calculated and were presented as median values or percentages. The Mann–Whitney test or chi‐squared test was used to assess group differences. For the pairwise comparisons and correlation analysis, *P* < 0.05 was considered to be statistically significant. To correct for the multiple comparisons, Bonferroni correction was used. Point‐biserial correlations were used to examine the relations of the chief complaints of SCD (*plus*) to age and education, respectively. In addition, given studied population is located in the rural area of Beijing, and the proportion of participants with low‐level education is high, we also set “6 years” (the year when the primary school is completed) as the cutoff to examine whether primary education is an important time point. For this reason, we also dichotomized the groups and performed group comparisons. Since few studies have reported the relationship between the SCD (*plus*) subgroups and age, in addition to the Point‐biserial correlation between age and SCD (*plus*) complaints, we further observed this relationship and conducted post hoc analyses by dividing the population into two groups with every 5‐year increment, three of which were also 25, 50, and 75 percentile of age. To examine the potential risk factors for different SCD (*plus*) subgroups, we performed binary logistic regression analysis, using “age, gender, education years, and SCD (*plus*) complaints, including SCD‐C, SCD‐F, and SCD‐P” as the independent variables, and “diagnosis” as the dependent variable. In addition, odds ratios (ORs) were calculated for each variable, *P* < 0.05 was required for variables to *be* in the model. Finally, we conducted Spearman correlations between single scores and total score of SCD‐Q9, and the complaints in SCD (*plus*) and its subgroups.

## Results

### Neuropsychological assessment scores in NC, SCD (*plus*) and its subgroups

In the end, 814 subjects completed the comprehensive neuropsychological assessments. Participant information was detailed in our previous publication of the Beijing SCD prevalence in 2017.^34^

**Figure 1:**
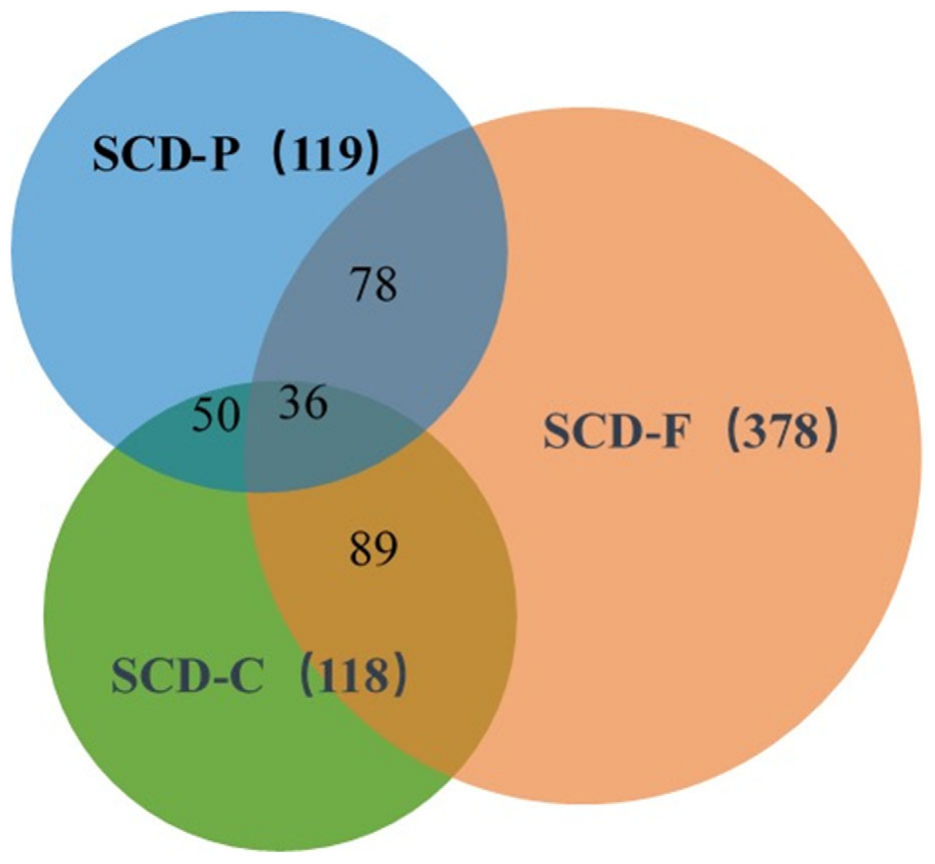
The composition and overlap of the SCD (*plus*) subgroups. SCD‐C, subjective cognitive decline‐concern; SCD‐F, subjective cognitive decline within the last 5 years; SCD‐P, subjective cognitive decline worse than their peers.

Eighty‐four NCs and 517 SCD (*plus*), including 118 SCD‐C, 378 SCD‐F, 119 SCD‐P, 372 SCD−, and 145 SCD+ were finally recruited in our study. There were some overlaps between the populations of subgroups (illustrated in Fig. 1).

The AVLT‐LR scores of the SCD (*plus*) and its subgroups were all lower than those of the NC group. However, according to corrected *P* value, only the difference of AVLT‐LR between the SCD‐P and NC group was significant (*P* < 0.008). The difference of AVLT‐LR between the SCD− and SCD+ group was not significant, with the value of latter slightly smaller than the former. Compared with the NC group, the SCD‐P group had lower scores of MoCA‐B (*P* < 0.008). The SCD+ group had lower scores of CDT and MoCA‐B compared to the SCD− group (*P* < 0.025). For the scores of STT and AFT, no significant differences were found between SCD (*plus*) and its subgroups versus NC, and SCD− versus SCD+ groups (see Table 1 for the results of all the groups).

**Table 1:** Scores of Neuropsychological assessments in NC, SCD (*plus*) and its subtypes.

Variables Percentile 50 (Percentile 25, 75)	Groups							*P*						
	NC	SCD (*plus*)	SCD‐C	SCD‐F	SCD‐P	SCD‐	SCD+	SCD (*plus*) versus NC	SCD‐C versus NC	SCD‐F versus NC	SCD‐P versus NC	SCD‐ versus NC	SCD+ versus NC	SCD‐ versus SCD+
AVLT‐LR	5 (4, 7)	5 (4, 6)	4 (4, 6)	5 (4, 6)	4 (3.6)	5 (4, 6)	5 (4.6)	0.012	0.009	0.037	0.001	0.020	0.017	0.682
MoCA‐B	23 (20, 25)	23 (20, 24)	22 (20, 24)	23 (20, 24)	21 (19, 24)	23 (21, 24)	22 (20, 24)	0.255	0.058	0.425	0.003	0.573	0.028	0.006
CDT	23 (20, 25.5)	23 (21, 25)	23 (20, 25)	23 (21, 25.625)	22 (20, 24.5)	23 (21, 26)	23 (20, 24)	0.348	0.912	0.294	0.347	0.148	0.676	0.009
STT	180.5 (147.25, 208.75)	180 (147, 210)	181 (152.5, 203.75)	180 (149.5, 210)	190 (147.75, 211.25)	180 (146, 215)	185 (160.5, 205.5)	0.991	0.937	0.978	0.590	0.878	0.626	0.454
AFT	16 (13, 18)	15 (13, 18)	15 (13, 17.5)	15 (13, 18)	15 (13, 18)	16 (13, 18)	15 (13, 17)	0.369	0.418	0.333	0.374	0.551	0.162	0.206

### Demographic characteristics of the three subgroups of SCD (*plus*)

For age, to test its relations to the chief complaints, point‐biserial correlations were used. The results showed that no significant correlations were found for SCD‐C (*r* = −0.065, *P* = 0.141), SCD‐F (*r* = −0.009, *P* = 0.843), and SCD‐P (*r* = −0.016, *P* = 0.715). The results of the post hoc analyses demonstrated that there was only an aging effect for SCD‐C group using 74 or 75 years old as the cutoff age (also see the scatterplots of age and Tables S1 and S2 in the appendix).

For education years, we also conducted point‐biserial correlation. The results showed that only SCD‐P group showed a significant correlation with education years (*r* = −0.184, *P* < 0.001), and no correlation was found for SCD‐C (*r* = −0.059, *P* = 0.182) and SCD‐F (*r* = 0.076, *P* = 0.084) groups. By using “6 years” as the cutoff, we also found individuals with lower education level felt that their cognitive performances were worse than their peers (SCD‐P; *P* < 0.05).

**Figure 2:**
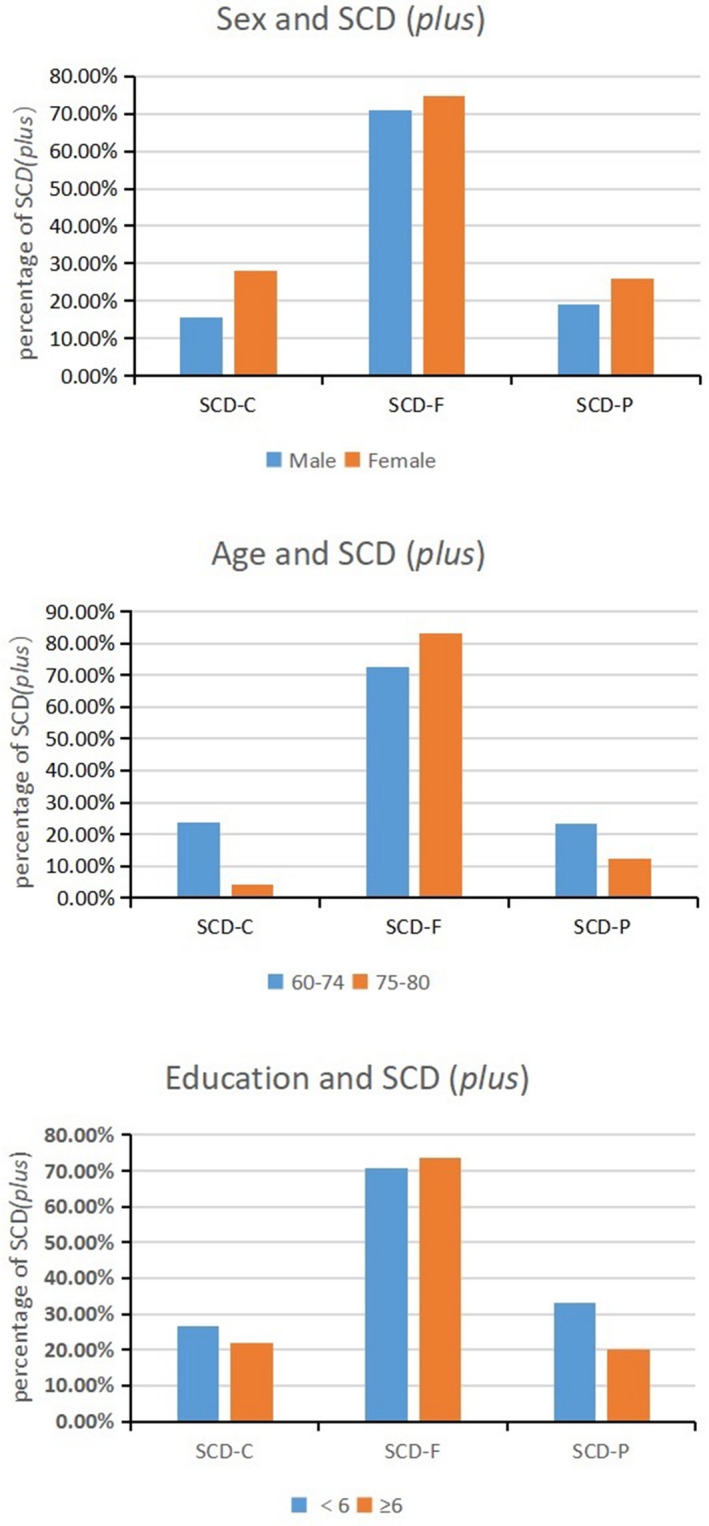
Demographic characteristics of SCD (*plus*) subgroups. SCD (*plus*), subjective cognitive decline (*plus*); SCD‐C, subjective cognitive decline‐concern; SCD‐F: subjective cognitive decline within the last 5 years; SCD‐P, subjective cognitive decline worse than their peers.

In addition, females were more concerned about their cognitive decline than males. No significant differences of age, gender, and education years were found in the SCD‐F group (see Table 2 and Fig. 2 for more details).

**Table 2:** Age, gender, and education effect in three subgroups of SCD (*plus*).

Variables	Groups			*P* (SCD‐C)	*P* (SCD‐F)	*P* (SCD‐P)
	SCD‐C *n* (%)	SCD‐F *n* (%)	SCD‐P *n* (%)			
Age				Younger vs elderly		
60–74	117 (23.7)	358 (72.6)	116 (23.5)	0.026	0.248	0.210
75–80	1 (4.2)	20 (83.3)	3 (12.5)			
Gender				Male vs female		
Male	34 (15.7)	153 (70.8)	41 (19.0)	0.001	0.322	0.065
Female	84 (27.9)	225 (74.8)	78 (25.9)			
Education (years)				Lower vs higher education level		
<6	31 (26.5)	83 (70.9)	39 (33.3)	0.282	0.547	0.003
≥6	87 (21.8)	295 (73.8)	80 (20.0)			

SCD (*plus*), subjective cognitive decline (*plus*). SCD‐C, subjective cognitive decline‐concern; SCD‐F, subjective cognitive decline within the last 5 years; SCD‐P, subjective cognitive decline worse than their peers.

### Affecting factors of three SCD (*plus*) subgroups

For the SCD‐C (Table 3), gender and SCD‐P were in the final regression equation, suggesting female as the independent risk factors for SCD‐C (OR: 2.023, 95%CI = 1.255–3.261). It also showed that individuals who had complaints of SCD‐P were more likely to report SCD‐C (Table 3, OR: 3.468, 95%CI = 2.183–5.510). Age, education years, and SCD‐F did not show any effect on SCD‐C.

**Table 3:** Affecting factors of SCD‐C.

Variables	*B*	SE	Wald	OR	95%CI	*P*
SCD‐P	1.244	0.236	27.707	3.468	(2.183, 5.510)	<0.001
SCD‐F	0.283	0.255	1.236	1.327	(0.806, 2.186)	0.266
Gender	0.705	0.244	8.371	2.023	(1.255, 3.261)	0.004
Age	−1.907	1.035	3.393	0.149	(0.020, 1.130)	0.065
Education	0.146	0.265	0.304	1.158	(0.688, 1.948)	0.581

SCD‐C, subjective cognitive decline‐concern; SCD‐P, subjective cognitive decline worse than their peers; SCD‐F, subjective cognitive decline within the last 5 years; OR: Odds ratios; CI, confidential interval.

Additionally, we found that individuals who reported SCD‐P were less likely to report SCD‐F as it is demonstrated by their negative association (Table 4, OR: 0.583, 95%CI = 0.367–0.928).

**Table 4:** Affecting factors of SCD‐F.

Variables	*B*	SE	Wald	OR	95%CI	*P*
SCD‐P	−0.539	0.237	5.183	0.583	(0.367, 0.928)	0.023
SCD‐C	0.291	0.255	1.302	1.338	(0.811, 2.206)	0.254
Gender	0.244	0.214	1.299	1.276	(0.839, 1.942)	0.254
Age	0.622	0.562	1.222	1.862	(0.619, 5.604)	0.269
Education	0.154	0.249	0.382	1.166	(0.716, 1.900)	0.537

SCD‐C, subjective cognitive decline‐concern; SCD‐F, subjective cognitive decline within the last 5 years; SCD‐P, subjective cognitive decline worse than their peers; OR, Odds ratios; CI, confidential interval.

Moreover, it showed that longer education years was a protective factor of SCD‐P (Table 5, OR:0.535, 95%CI = 0.325–0.882), but it did not have any effect on SCD‐F. Also, age and gender did not show any effect on SCD‐P and SCD‐F.

**Table 5:** Affecting factors of SCD‐P.

Variables	*B*	*SE*	Wald	OR	95%CI	*P*
SCD‐C	1.251	0.237	27.871	3.493	(2.195, 5.557)	<0.001
SCD‐F	−0.539	0.237	5.192	0.583	(0.367, 0.927)	0.023
Gender	0.100	0.240	0.174	1.105	(0.691, 1.769)	0.676
Age	−0.351	0.639	0.302	0.704	(0.201, 2.461)	0.582
Education	−0.625	0.255	6.018	0.535	(0.325, 0.882)	0.014

SCD‐C, subjective cognitive decline‐concern; SCD‐F, subjective cognitive decline within the last 5 years; SCD‐P, subjective cognitive decline worse than their peers; OR, Odds ratios; CI, confidential interval.

### Scores of SCD‐Q9 in NC, SCD (*plus*) and its subgroups

The results showed that the total scores of SCD‐Q9 increased in the order of NC → SCD− → SCD+. All the single and total scores of the SCD‐Q9 were lower in the NC group than those in SCD (*plus*) and its subgroups (*P* < 0.001). Moreover, several single scores and total score of SCD‐Q9 were higher in the SCD+ than the SCD− group (*P* < 0.001), except SCD items 4 (*P = *0.627), 5 (*P = *0.242), and 8 (*P = *0.094) (see Table S3 for more details in the appendix).

Finally, according to the corrected *P* value (*P* < 0.125), we found significantly positive correlations between the total and all the single scores of SCD‐Q9 and SCD (*plus*) complaints, except SCD‐F. Also, our results illustrated that the correlation coefficient value between the total score of SCD‐Q9 and SCD‐F (*r* = 0.179) was smaller than both the SCD‐P (*r* = 0.405) and SCD‐C (*r* = 0.387) (see Table S4 for more details in the appendix).

## Discussion

For the first time, this study reported the demographic characteristics and affecting factors of SCD (*plus*) based on a large Chinese community population. To our best knowledge, this is also the first study that revealed the presence of early cognitive impairment in population with different SCD (*plus*) subgroups in China, according to the diagnostic framework.^7^ Finally, this is the first attempt to analyze the correlation between SCD (*plus*) complaints and the scores of SCD‐Q9.

In our study, the SCD‐P showed lower scores of AVLT‐LR than NC group (*P* < 0.008), suggesting that memory impairment has already occurred at this preclinical stage of AD. A few studies have shown that delayed recall might be a sensitive indicator of early cognitive impairment.^35^, ^36^ Kielb et al. found that SCD (*plus*) had more severe functional impairment, including reduced episodic memory and poorer performance on psychomotor speed and language, compared to the non‐SCD (*plus*) group.^37^ Thus, to better identify SCD (*plus*), the tests for episodic memory and delayed recall may be very useful and should be included as part of the neuropsychological assessments in the future studies.^38^

Our results demonstrated that in addition to AVLT‐LR, MoCA‐B may be a sensitive screening tool for SCD (*plus*) as well. Previous epidemiological studies showed that MoCA was superior to MMSE as a brief and feasible assessment, particularly in discerning earlier stages of cognitive decline.^38^ MoCA‐B is a modified version of the MoCA, and was validated to be a sensitive assessment of MCI and dementia for elderly subjects with low education.^39^, ^40^, ^41^ We found that SCD (*plus*) subgroup had lower scores of MoCA‐B than those in the NC group, suggesting that the global cognition impairment was already present from the stage of SCD (*plus*). Also, SCD+ group scored lower than SCD−, showing that the global cognition deteriorated as the number of complaints increased.

CDT is one of the most commonly used cognitive screening tools for dementia in clinics because of its handiness^42^, ^43^ as well as its sensitivity to the global cognitive function.^42^ A variety of other cognitive functions including orientation, selective and persistent attention, auditory comprehension, verbal memory, numerical knowledge, visual memory and reconstruction, visuospatial organization, and motor performance can be assessed by this simple test.^44^, ^45^ Many studies have investigated the accuracy of CDT for evaluating the level of cognitive impairment caused by dementia, using qualitative approaches together with either quantitative or semi‐quantitative methods.^46^ In our study, we used the version of CDT‐30 scoring system.^29^ Considering time‐consuming and consistency, only quantitative methods were used in this study, and small but significant cognitive decline was found in our SCD+ group. This is consistent with a previous study,^37^ suggesting that CDT can be used as a simple screening tool for early stage of AD, such as SCD (*plus*).^47^

The Subjective Cognitive Impairment Cohort (SCIENCe) study^48^ claimed that SCD is a heterogeneous group. Our results showed that the three subgroups differed in predicting the neuropsychological changes of SCD (*plus*). Only people in the SCD‐P group, who felt their performance was not as good as their peers, showed lower scores of AVLT‐LR than those in the NC group. Again, SCD+ had lower scores in the tests of MoCA and CDT than the SCD− group, but not in the AVLT. A previous study reported that individuals with SCD‐C had a higher risk of progressing to dementia.^49^ and we did find that SCD‐C group had a lower scores of AVLT‐LR test compared to the NC group, even though the difference was not significant (*P* > 0.008). Surprisingly, we found individuals who had complaints of SCD‐F were less likely to report SCD‐P. Although there is an overlapping between the two groups, we expect that the negative effect would be more enhanced when the overlapping was removed. In other words, our participants who reported cognition decline within the last 5 years did not feel their memory was worse than their peers, and vice versa. This could be attributed to the following possible reasons: (1) the 5‐year time frame might not be a specific indicator of SCD (*plus*) as a majority of our subjects reported SCD‐F (378/517). Also, both questions in SCD‐Q9 regarding the onset of SCD (items 3 and 8) had high correlations with SCD‐F, which may indicate that the timescale of SCD plus’s progression may not be a clear concept to our participants. Finally, correlations between SCD‐Q9 and the complaints showed that compared with SCD‐C and SCD‐P, SCD‐F had the lowest coefficient, again suggesting that "5‐year" may not be a key discrimination point for the diagnostic criteria^7^; (2) it is possible that some participants in our study consider "worse than peers" is a shameful issue,^50^ especially in rural areas in China although they admitted their cognitive decline in the recent years. Additionally, we found that individuals who had complaints of SCD‐C were more likely to report SCD‐P, demonstrated by the high positive OR value. This may be explained by the fact that approximately 50% of SCD‐P and SCD‐C individuals reported those items as the chief complaints, suggesting that feeling the cognitive performance "worse than their peers" for some people might be accompanied by the concern about their cognitive decline.

Only few studies focused on the demographic characteristics of SCD (*plus*).^20^ A previous study proposed that gender should be considered in the interpretation of the cognitive assessment of SCD.^51^ Our study demonstrated that females expressed concerns more than males at similar ages, which was consistent with results from previous studies.^6^, ^13^ Also, the younger group (<75 years old) expressed more worries about their cognitive condition than the older group (≥75 years old). However, there was only one participant in the older group. Thus, more senior participants should be recruited to check whether this finding is valid. Individuals with lower level education were more likely to report that they felt their performance was not as good as their peers, which was consistent with a study based in Japan, and they also concluded less education was associated with more subjective neurocognitive complaints.^14^

SCD has recently become the focus of research since many studies have demonstrated that it was highly possible for SCD individuals who fulfill the criterion of SCD (*plus*) to progress into preclinical AD.^7^ Thus, it is important to identify a cognitive questionnaire to reflect SCD (*plus*) complaints.^19^, ^20^, ^21^ In our study, we found the total score and all the single scores of SCD‐Q9 in the SCD (*plus*) and its subgroups were higher than those in the NC group. Moreover, our results illustrated that the total score and several single scores of SCD‐Q9 in SCD‐ group were lower than those in the SCD+ group, suggesting that SCD‐Q9 may be able to partially correspond to these subjective complaints. Also, it was assuring to learn that there was high consistency between the similar questions in both chief complaints and SCD‐Q9, (between SCD‐3 vs. SCD‐C and SCD‐8 vs. SCD‐F, respectively). Taken together, these findings implicate that SCD‐Q9 may be sensitive to be used as a discriminating tool of SCD (*plus*).

### Implications and limitations

Different complaints of SCD (*plus*) may have different implications among the aged population. It usually can be classified into age‐related physiological alterations and/or pathological symptoms. The findings highlighted in this study illustrated the need for caution when selecting SCD measures, and the potentials of using SCD's chief complaints to inform the underlying neurobiology.^52^

The limitations of this study include: (1) although the sample size was larger than the other studies,^49^, ^51^ our sample size maybe still limited. The population of the subgroups is even smaller and there were overlaps among them, albeit that we analyzed their associations. Investigations with larger sample sizes are needed in the future; (2) Our study is a cross‐sectional survey and follow‐up studies should be performed to further confirm the results; (3) the current study lacks the completeness of amyloid *β*‐protein (A*β*)‐PET, ApoE ε4, cerebrospinal fluid tau or A*β* examinations, and the diagnosis of SCD (*plus*) was not validated by the other tests; (4) other related biomarkers and imaging approaches need to be applied to gain more understandings of SCD (*plus*); (5) although the results regarding the subgroups of SCD (*plus*) features may be culture specific and further studies are needed, we suggest that cautions should be taken for researches involving various ethnicities and different levels of education, especially when large data repositories contributing from China are used in the future.

In summary, this study emphasized the importance of neuropsychological objective assessment for individuals with SCD (*plus*). SCD (*plus*) chief complaints may need to be expounded according to demographic factors including age, gender, and years of education. Finally, SCD (*plus*) as features of preclinical AD may be partially reflected by SCD‐Q9, but their associations still call for further studies.

## Conflict of Interest

There is no conflict of interest.

## Supporting information


**Figure S1.** The scatterplots of different age associated with three subgroups of SCD (*plus*) (appendix).

**Table S1.** Group comparison of SCD (plus) subgroups based on different cut‐off ages.
**Table S2.** Group comparison of SCD (plus) subgroups based on different cut‐off ages.
**Table S3.** Scores of SCD‐Q9 in the NC, SCD (plus) and its subgroups.
**Table S4.** Correlations between SCD‐Q9 scores and the complaints of SCD (plus).
