# Supplementary figures and images for: Demographic characteristics and neuropsychological assessments of subjective cognitive decline (SCD) (plus)
Source: Ann Clin Transl Neurol. 2020 Jun 26;7(6):1002–12. doi: 10.1002/acn3.51068 (PMC7317645; doi:10.1002/acn3.51068)

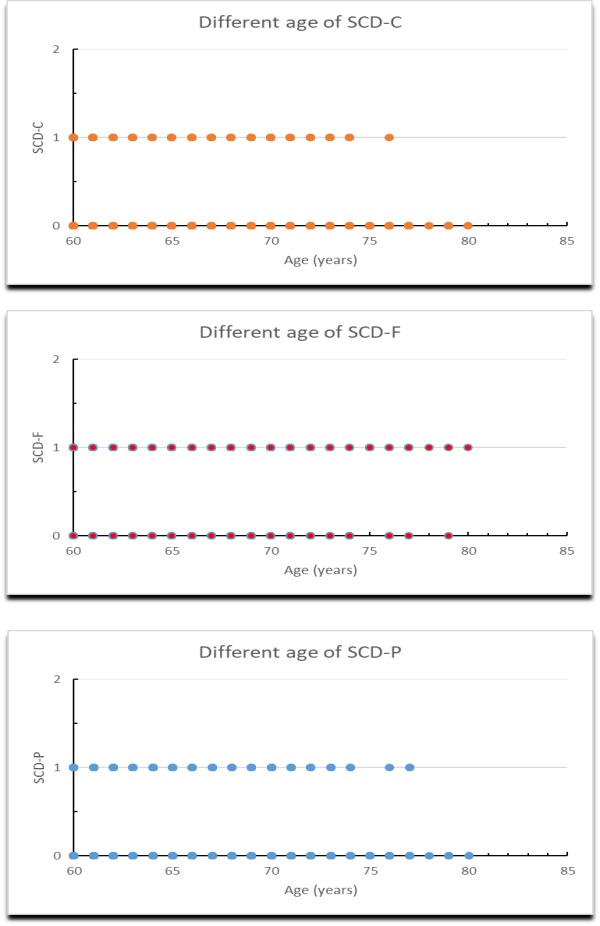

Supplement: Supplementary file 1 — Figure S1. The scatterplots of different age associated with three subgroups of SCD (plus) (appendix). [file ACN3-7-1002-s001.tif]
