# Supplementary material for: Demographic characteristics and neuropsychological assessments of subjective cognitive decline (SCD) (plus)
Source: Ann Clin Transl Neurol. 2020 Jun 26;7(6):1002–12. doi: 10.1002/acn3.51068 (PMC7317645; doi:10.1002/acn3.51068)

**APPENDIX**

**Table 1. Group comparison of SCD (*plus*）subgroups based on different cut-off ages**

| Cut-off age | Groups | | | *P*  (SCD-C) | *P*  (SCD-F) | *P*  (SCD-P) |
| --- | --- | --- | --- | --- | --- | --- |
|  | SCD-C  n (%) | SCD-F  n (%) | SCD-P  n (%) | *Younger and older group comparisons using different cut-off ages* | | |
| *Use 25 Percentile:*  *61 years* |  |  |  | 0.505 | 0.851 | 0.498 |
| 60-61 | 27(25.2) | 79(73.8) | 22(20.6) |  |  |  |
| 62-80 | 91(22.2) | 299(72.9) | 97(23.7) |  |  |  |
| *Use 50 Percentile:*  *65 years* |  | | | 0.465 | 0.585 | 0.404 |
| 60-65 | 61(24.2) | 187(74.2) | 62(24.6) |  |  |  |
| 66-80 | 57(21.5) | 191(72.1) | 57(21.5) |  |  |  |
| *Use 75Percentile:*  *69 years* |  |  |  | 0.421 | 0.863 | 0.883 |
| 60-69 | 91(23.7) | 280(72.9) | 89(23.2) |  |  |  |
| 70-80 | 27(20.3) | 98(73.7) | 30(22.6) |  |  |  |

**SCD (*plus*):** subjective cognitive decline (*plus*). **SCD-C:** subjective cognitive decline-concern. **SCD-F:** subjective cognitive decline- within the last 5 years. **SCD-P:** subjective cognitive decline- worse than their peers.

**Table 2. Group comparison of SCD (*plus*）subgroups based on different cut-off ages**

| Cut-off age | Groups | | | *P*  (SCD-C) | *P*  (SCD-F) | *P*  (SCD-P) |
| --- | --- | --- | --- | --- | --- | --- |
|  | SCD-C  n (%) | SCD-F  n (%) | SCD-P  n (%) |  |  |  |
| *64 years* |  | | | 0.413 | 0.296 | 0.607 |
| 60-64 | 55(24.6) | 169(75.4) | 54(24.1) |  |  |  |
| 65-80 | 63(21.5) | 209(71.3) | 65(22.2) |  |  |  |
| *74 years* |  | | | 0.026* | 0.248 | 0.210 |
| 60-74 | 117(23.7) | 358(72.6) | 116(23.5) |  |  |  |
| 75-80 | 1(4.2) | 20(83.3) | 3(12.5) |  |  |  |

*: significant result with *p*<0.05. **SCD (*plus*):** subjective cognitive decline (*plus*). **SCD-C:** subjective cognitive decline-concern. **SCD-F:** subjective cognitive decline- within the last 5 years. **SCD-P:** subjective cognitive decline- worse than their peers.

**Table 3. Scores of SCD-Q9 in the NC, SCD (*plus*) and its subgroups**

| Variables | Groups | | | | | | |
| --- | --- | --- | --- | --- | --- | --- | --- |
|  | NC  Percentile  50  (Percentile 25，75) | SCD (*plus*)  Percentile  50  (Percentile 25，75) | SCD-C  Percentile  50  (Percentile 25，75) | SCD-F  Percentile  50  (Percentile 25，75) | SCD-P  Percentile  50  (Percentile 25，75) | SCD-  Percentile  50  (Percentile 25，75) | SCD+  Percentile 50  (Percentile 25, 75) |
| SCD-1 | 0(0,0) | 1(0,1) | 1(1,1) | 1(0,1) | 1(1,1) | 1(0,1) | 1(1,1) |
| SCD-2 | 0(0,0) | 0(0,1) | 1(0,1) | 0(0,1) | 1(0,1) | 0(0,1) | 1(0,1) |
| SCD-3 | 0(0,0) | 1(0,1) | 1(1,1) | 1(0,1) | 1(1,1) | 1(0,1) | 1(1,1) |
| SCD-4 | 0(0,0) | 0(0,0.5) | 0(0,0.5) | 0(0,0.5) | 0(0,0.5) | 0(0,0.5) | 0(0,0.5) |
| SCD-5 | 0(0,0) | 0(0,0.5) | 0.5(0,0.5) | 0(0,0.5) | 0.5(0,0.5) | 0(0,0.5) | 0(0,0.5) |
| SCD-6 | 0(0,0) | 0(0,1) | 1(0,1) | 0(0,1) | 1(0,1) | 0(0,1) | 1(0,1) |
| SCD-7 | 0(0,0.5) | 0.5(0,0.5) | 0.5(0.5,0.5) | 0.5(0,0.5) | 0.5(0,0.5) | 0.5(0,0.5) | 0.5(0,0.5) |
| SCD-8 | 0(0,1) | 1(1,1) | 1(1,1) | 1(1,1) | 1(1,1) | 1(1,1) | 1(1,1) |
| SCD-9 | 0(0,1) | 1(0,1) | 1(1,1) | 1(0,1) | 1(1,1) | 1(0,1) | 1(1,1) |
| SCD-Total | 0  (0,2.5) | 5.0  (3.0,6.75) | 6.0  (3.875,7.5) | 4.5  (2.5,6.5) | 6.5  (3.5,7.5) | 4.5  (2.5,6.5) | 6.0  (3.5,7.25) |

**NC:** normal control; **SCD (*plus*):** subjective cognitive decline (*plus*); **SCD-Q9:** subjective cognitive decline-questionnaire 9; **SCD-C:** subjective cognitive decline-concern; **SCD-F:** subjective cognitive decline- within the last 5 years; **SCD-P:** subjective cognitive decline- worse than their peers; **SCD-:** presented ≤ 3 of SCD (*plus*) features; **SCD+:** presented > 3 of SCD (*plus*) features. For SCD-Q9, the number following SCD- represents the question number.of SCD- Q 1 to 9.

**Table 4. Correlations between SCD-Q9 scores and the complaints of SCD（*plus*）**

| Variables | SCD (*plus*) | SCD-C | SCD-F | SCD-P |
| --- | --- | --- | --- | --- |
|  | r  （*p*） | r  （*p*） | r  （*p*） | r  （*p*） |
| SCD-1 | .343  (<0.001) | .323  （<0.001） | .206  （<0.001） | .337  （<0.001） |
| SCD-2 | .217  (<0.001) | .248  （<0.001） | .092  （0.024） | .306  （<0.001） |
| SCD-3 | .382  (<0.001) | .370  （<0.001） | .259  （<0.001） | .371  （<0.001） |
| SCD-4 | .193  (<0.001) | .181  （<0.001） | .036  （0.381） | .178  （<0.001） |
| SCD-5 | .184  (<0.001) | .194  （<0.001） | -.006  （0.884） | .185  （<0.001） |
| SCD-6 | .204  (<0.001) | .226  (<0.001） | .063  （0.121） | .283  （<0.001） |
| SCD-7 | .216  (<0.001) | .262  (<0.001) | .102  （0.013） | .206  （<0.001） |
| SCD-8 | .354  (<0.001) | .290  (<0.001) | .225  （<0.001） | .298  （<0.001） |
| SCD-9 | .302  (<0.001) | .286  (<0.001) | .175  （<0.001） | .269  （<0.001） |
| SCD-Total | .386  (<0.001) | .387  (<0.001) | .179  （<0.001） | .405  （<0.001） |

**NC:** normal control; **SCD (*plus*):** subjective cognitive decline (*plus*); **SCD-Q9:** subjective cognitive decline-questionnaire 9; **SCD-C:** subjective cognitive decline-concern; **SCD-F:** subjective cognitive decline- within the last 5 years; **SCD-P:** subjective cognitive decline- worse than their peers; For SCD-Q9, the number following SCD- represents the question number.of SCD- Q 1 to 9.


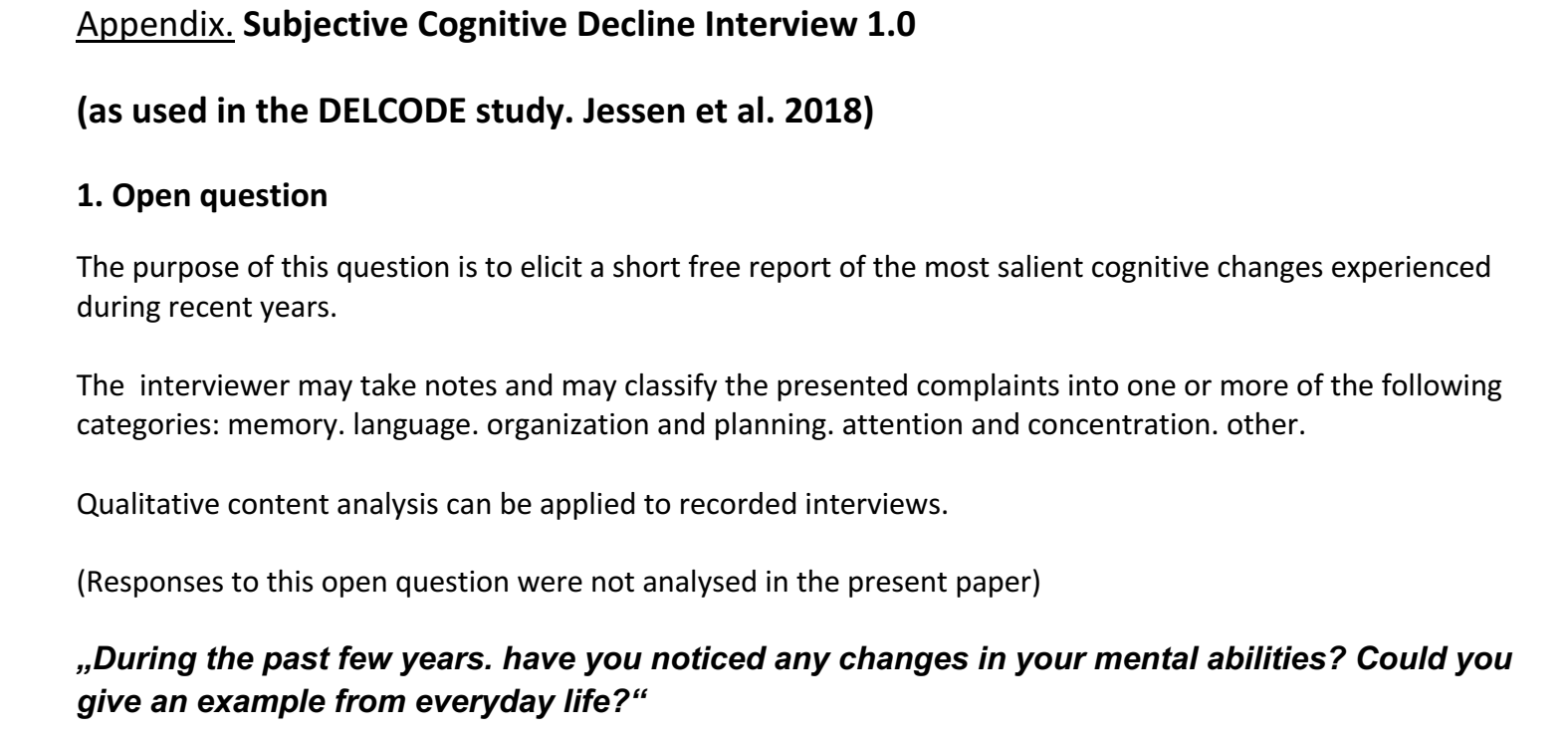

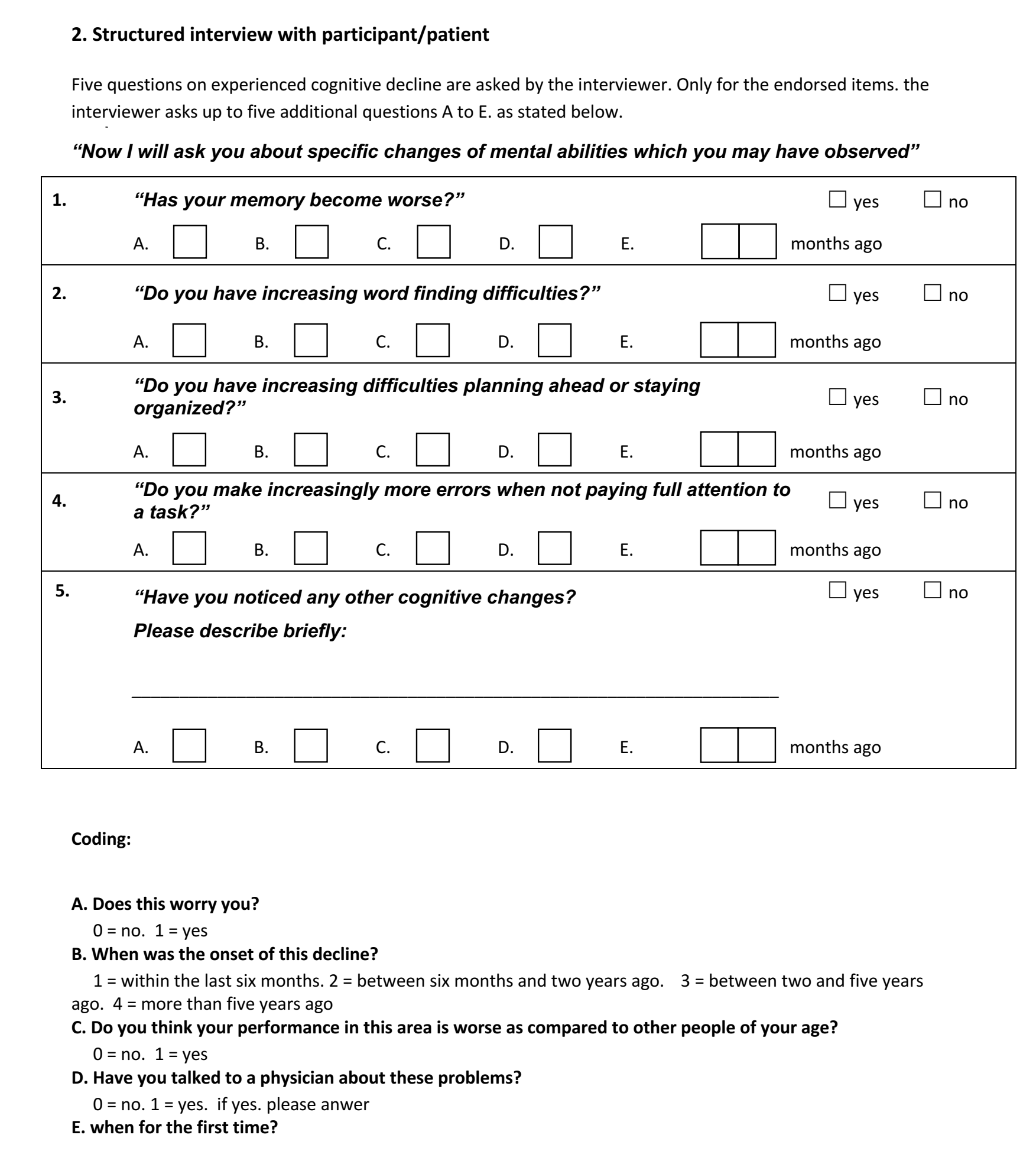

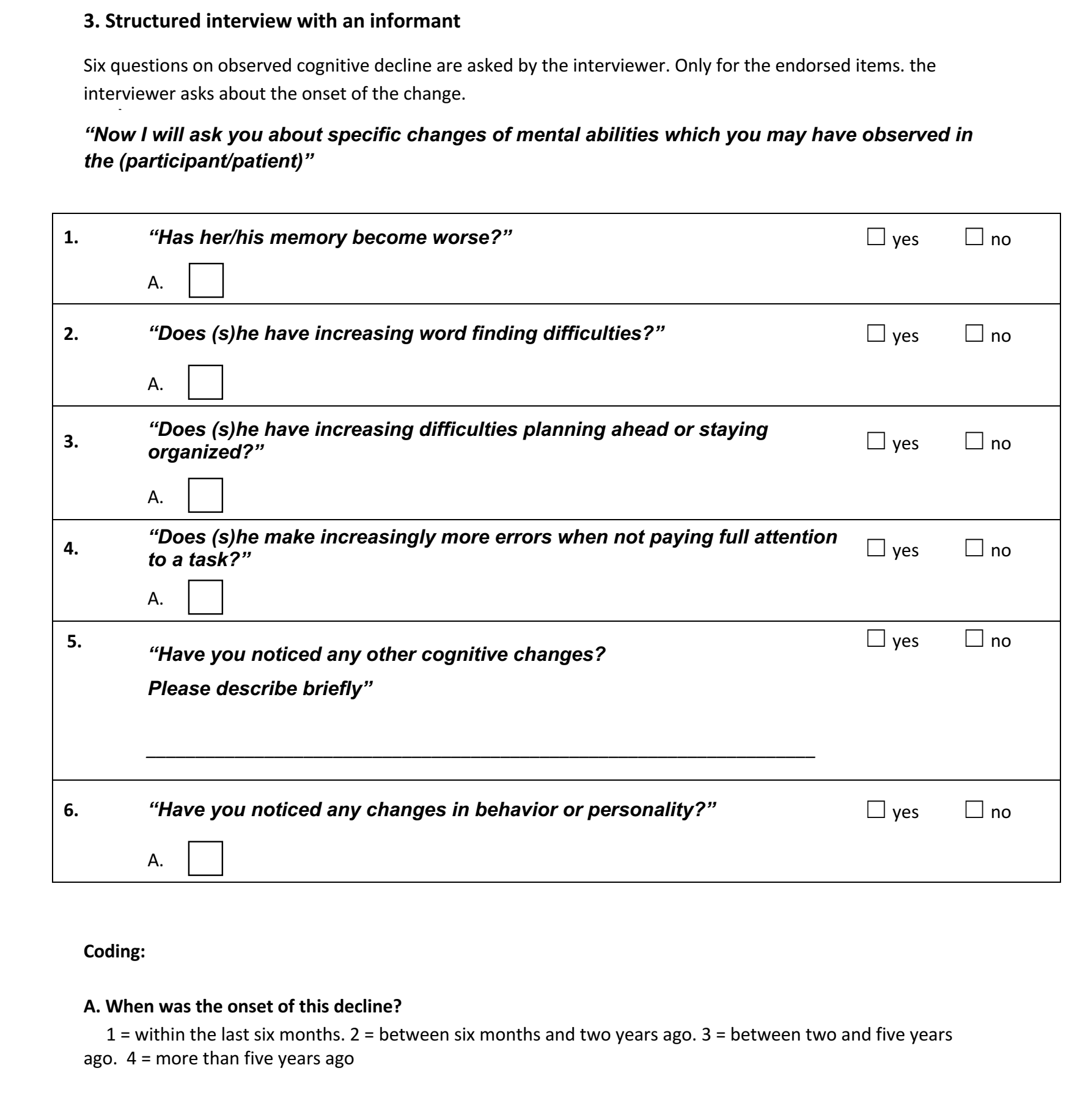

Supplement: Supplementary file 2 — Table S1. Group comparison of SCD (plus) subgroups based on different cut‐off ages. Table S2. Group comparison of SCD (plus) subgroups based on different cut‐off ages. Table S3. Scores of SCD‐Q9 in the NC, SCD (plus) and its subgroups. Table S4. Correlations between SCD‐Q9 scores and the complaints of SCD (plus). [file ACN3-7-1002-s002.docx]
